# Quantitative assessment of the jawbone quality classification: A meta-analysis study

**DOI:** 10.1371/journal.pone.0253283

**Published:** 2021-06-16

**Authors:** Keren Shemtov-Yona

**Affiliations:** 1 Faculty of Mechanical Engineering, Technion, Haifa, Israel; 2 The Maurice and Gabriela Goldschleger School of Dental Medicine, Department of Oral Biology, Tel Aviv University, Tel Aviv, Israel; Nanjing Medical University, CHINA

## Abstract

**Aim:**

Bone quality is evaluated using bone density for qualitative classification, a characteristic that may be delicate to evaluate. Contemporary implantology that relies on modern measurement techniques, needs a more quantitative estimate of the bone quality.

**Materials and methods:**

PubMed and EMBASE databases were searched with no time restriction. Clinical and radiographic studies reporting on alveolar ridge dimensions and its parameters in different areas of the dentate and edentulous jaws were included. A meta-analysis was performed using random effect models to report a combined mean for alveolar ridge and its parameters. Meta regression statistical tests were performed in order to identify differences in those outcome parameters.

**Results:**

30 studies were included. The majority of the selected studies (total of 27) used live human subjects and CBCT to analyze alveolar ridge dimensions and its parameters. Using the combined mean obtained from the meta-analysis, a typical portrait of the alveolar ridge was constructed, and a geometrically based quantitative bone classification proposed. The quantitative classification was found to match the existing qualitative classification.

**Conclusion:**

A geometry-based analysis was constructed that yields valuable insights on the bone type based on its components and on the dynamics of the dentate / edentulous states.

## 1. Introduction

Dental implants are the most popular and predictable modern solution for missing teeth. The early and long-term success of dental implants depends largely on the alveolar bone quantity and quality during implant placement [1, 2]. Poor bone quality and quantity are considered as a risk factor for biological complications of the implant, associated with lack of primary stability and impaired healing / osseointegration, that can lead to early implant loss [2].

The external architecture of the dentate or edentulous alveolar bone and its volume are primarily evaluated during treatment planning for dental implants. The external and internal architecture of bone controls virtually every facet of the practice of implant dentistry from implant design selection, surgical approach, healing time, type of future prosthetic reconstruction etc.

Lekholm and Zarb [3] listed four types of bone quality found in the *anterior regions* of the jawbone. This classification, widely used in modern implant dentistry, is essentially *qualitative* and defines bone quality based on the relationship between the compact cortical and the trabecular bones, as follows.

Quality 1 is for homogeneous compact bone, and Quality 2 describes a thick layer of compact bone surrounding a core of dense trabecular bone. Quality 3 refers to a thin layer of cortical bone surrounding dense trabecular bone, and Quality 4 corresponds to a thin layer of cortical bone surrounding a core of low-density trabecular bone.

A correlation between bone quality and implant failure was found in a recent systematic review [4], according to which the survival percentage of surface treated implants inserted to Quality 1 was 97.6%, Quality 2, 96.2%; Quality 3, 96.5%; and a reduction in implant’s survival rate in Quality 4 bone of 88.8% the mean follow-up was 53.7 months. This result clearly emphasizes the importance of bone quality (and quantity) for implant survival, and the correlation to the Lekholm and Zarb’s [3] classification.

In 1988, Misch proposed four (D1-D4) bone type groups based on macroscopic cortical and trabecular bone characteristics (density) located in edentulous areas of the maxilla and mandible (see Resnik and Misch [5]). This work indicated that different bone types may often be encountered in different regions of the mouth. D1 bone type is considered rare, and mostly found in the anterior mandible. D2 bone type is the most common bone type, and it can be found in most areas of the mandible while D3 bone type is frequent in the anterior maxilla. Finally, D4 bone type is most often identified in the posterior maxilla.

These *qualitative* classifications can be further extended, and to some extent quantified, by considering the ratio between cortical to cancellous bone volume, as assessed in a routine CT scan. In that spirit, Chatvaratthana et al. [6] reported that a ratio in excess of 0.75 (75–100% cortical bone) corresponds to D1 bone type, 0.5–0.75 (50–75% cortical bone) to D2 bone type, 0.25–0.5 (25–50% cortical bone) to D3 bone type and D4 bone type contains less than 0.25 (0–25% cortical bone). This classification does not take into consideration the total bone volume and does not differentiate between the different areas of the maxilla and the mandible.

Despite the wide use of the above-mentioned bone classifications, be it pre-operative or operative, they are all essentially subjective, a point raised by Brunski in [7], and rely mostly on bone density or to the surgeon’s perception during drilling the implant osteotomy [5, 8]. In addition, the classification lacks the clear quantitative information on the relative contribution of each component of the ridge, the cortical bone component (buccal plate/lingual plate/palatal plate) and the trabecular bone component on the anatomy of the ridge and thus the bone type group. Furthermore, geometrical variations are objective, straightforward and easier to measure whereas density maps are much more delicate to interpret, and prone to bias due to method variations, all the more so if only subtle local evolutions are to be determined.

The alveolar process is defined as part of the maxilla and mandible that forms around and supports the teeth. Its volume, shape and height are determined by the shape of the teeth (crown and root) they harbor [9]. The bony component of the alveolar process is comprised of outer plates, the facial (labial and buccal) surface and the lingual/palatal surface, these plates being made of thin cortical bone. The roots are covered by the attachment apparatus which includes the root cementum, periodontal ligament, and the alveolar bone proper (bundle bone) that is made of cortical bone as well. The area enclosed by the cortical bone plates is occupied by cancellous (spongy) bone [10, 11]. The cortical surfaces are in continuity with the body of the jaw and vary in thickness from one region of the jaw to the other.

Both in the mandible and in the maxilla, the facial plate is usually thin and tends to be thicker in the second and third molar area. The palatal/lingual plates of the alveolar process are usually thicker than the facial plate. Despite the basic similarity between the two jaws, the alveolar process in the mandible it is not as cancellous as that in the maxilla. The thin facial bone plate, most often seen in the anterior area of the mandible, its composed of mainly cortical dense compact bone as seen on the lingual plate [11, 12].

When teeth are lost, the portion of the alveolar process that supported the missing tooth will be subject to atrophic reduction, where the size of the ridge will become markedly reduced in both its horizontal and vertical dimensions [13, 14]. The percentage of vertical change was reported to be 11–22% at 6 months, while the percentage of horizontal change was reported to be 32% after 3 months, and 29–63% between 6 and 7 months [15, 16]. The horizontal resorption of the buccal component of the ridge was shown to reach as much as 56%, while lingual component reduction was reported to be much less, up to 30% [17]. In addition, the absolute amounts of tissue loss varied from one group of teeth to the next [15] and on the area of the jaw non-molar vs. molar areas [18].

The buccal bone plate’s original dimensions have a large effect on the degree of ridge resorption following tooth extraction. It was reported that bone plates that were less than 1 mm wide lost substantially more dimension (width and height) than wider plates, along with a more pronounced horizontal dimension loss [18–21]. This clear difference in post-extraction remodeling behavior relies on the histological characteristics of the bone plate, as the thin facial plate is predominantly composed of bundle bone that is functionally dependent on tooth presence and will thus completely resorb following tooth extraction or loss. This resorption will result in pronounced vertical reduction of the ridge height. For the wider lingual crest and more apical parts of the buccal plate, where the bone composition is lamellar, compact in nature and less affected by the bundle bone resorption, less changes are expected [22].

Araujo and Lindhe [23] evaluated dimensional alterations of the alveolar ridge that occurred following premolar tooth extraction in a dog model, by measuring the width of the facial and lingual plates 1mm, 3mm and 5 mm, from the crestal part, during 8 weeks after healing. The study showed that the facial plate width was reduced by up to 20% and the lingual plate narrowed by up to 15% in all examined bone levels.

Katranji et al. [24] performed a quantitative human cadaver head study to evaluate the differences in ridge anatomy in dentate and edentulous ridges. The facial and lingual / palatal plate thickness was evaluated. Differences in cortical bone width were observed in different areas of the jaw (e.g., anteriors, premolars, or molars), as well as between the different jaws (e.g., maxilla or mandible) and between dentate and edentulous jaws.

Two kinds of assessment methods exist for the bone quality and quantity. The direct measurement techniques include ex vivo studies (i.e., dry skulls or cadaver) or sample / biopsy retrieved for analysis from animals or human subjects, and in vivo studies done on live subjects. The indirect measurement techniques are based on radiographic imaging, such as CT or CBCT. These measurement techniques provide a three-dimensional depiction of bony structures and are considered to be an accurate diagnostic tool that, in addition to linear measurements, enables evaluation of the morphology, bone quality, and volume of the residual alveolar ridge. [25].

The present study aims firstly at defining and evaluating the thickness of the alveolar ridge, palatal/lingual and buccal cortical bone in various regions of the maxilla and mandible, dentate and edentulous, while trying to *systematically identify the different commonly accepted bone types*, based on quantitative geometric data published in the literature and gathered according to the PRIAMA guidelines. In addition, the study singles out the parameters of the alveolar ridge in both dentate and in edentulous states, that most influence the different bone types in different areas of the jaws using meta-analysis and meta regression statistical tests.

## 2. Materials and methods

In order to evaluate and identify the different alveolar ridge parameters, the study collected and analyzed data from the literature, using recommended tools and analysis methods for systematic reviews and meta-analyses [26, 27].

### 2.1 Patient, Exposure, Comparison, Outcome (PECO) questions

This study was performed using a PECO (Patient or Population, Exposure/Intervention, Control or Comparison, Outcome and Study design) framework. The *population* was defined as human patients (alive or cadaver), that were part of a study related to dental implants or orthodontic implant placement or anatomical studies. The *exposure* was anatomical factors including the impact of area (anterior, premolar, posterior), jaw (maxilla and mandible) and state (dentate and edentulous on *outcomes* associated with linear measurements of the alveolar ridge and its components / dimensions. The dimensions, measured in millimeters, were set as the outcome. In the eventuality of a comparative study between two or more groups, those groups were considered as outcomes irrespective of the conclusions of the study. A *comparison* was performed between the different anatomical parameters of the alveolar ridge in order construct different bone types based on geometrical factors and to evaluate their respective contribution to the classification.

### 2.2 Search strategies

An electronic search without any time restrictions was undertaken initially in the National Library of Medicine database (Medline) through its online site (PubMed), followed by searches in the EMBASE database.

The main terms used during the search are related to the study aim as “Edentulous” and/or “Dentate”, “Cortical (bone or wall), “Buccal” (or”Facial”) and/or “Palatal” and/or “Lingual” dimensions (or width or thickness) or alterations (or morphology), “Alveolar ridge” (or process) dimensions (or width/thickness) “Anterior” and/or “Premolar” and/or “Posterior” Maxilla/Mandible, “CBCT” and/or “CT” and/or “cadaver”. Additional Text terms as well as MeSH keywords and Emtree keywords specific for the study question are listed in the appendix section (S1 Table).

The date of the latest literature search was done in August 2020.

### 2.3 Study selection

#### 2.3.1 Inclusion/exclusion criteria

Eligibility criteria were as follows:

- Studies performing linear measurements for quantitative assessment (e.g., height, width) of the alveolar bone at dentate or edentulous sites or measuring distances from anatomical structures related to anatomical studies/orthodontics /implant dentistry/maxillofacial surgery/periodontology.
- Studies giving information on the study population (number of participants/sex/age) and addressing both adults and children over 13.
- Clinical studies using radiographic tools as CT/CBCT (type of sections), in-vivo clinical studies with a sample size greater than 5, in-vitro studies using human cadavers or dry skulls measuring linear distances in alveolar bone.
- Measurement’s data: Alveolar ridge width (RW), buccal cortical bone width (BW), and palatal/lingual bone width (PW/LW), all reported in millimeters. The included studies had to provide full information on which jaw the measurements were taken, which part of the jaw (anterior, premolar, or posterior) or tooth/site position in the jaw. In addition, the study had to include the exact location of the measurements from the top of the alveolar crest (in millimeters).
- Statistical data: Included only studies that used *parametric statistical tests* with normal distribution of the study population. The given results must be the mean, standard deviation and number of patients included in the study group.

The exclusion criteria were defined as:

- Experimental (animal) studies.
- Review articles.
- Studies of populations restricted to specific diseases or conditions.
- Intervention studies with case selection, or studies performing measurements prior to and/or post treatment, that might affect the alveolar bone dimensions (e.g. horizontal /vertical GBR, socket preservation, split-ridge bone augmentation or any type of surgical intervention aimed at changing bone ridge dimensions or components).

#### 2.3.2 Study selection process

Two independent observers analyzed the titles and abstracts of all identified reports. (K.S.Y and D.R) For the studies that appeared to meet the inclusion criteria, or for which there were insufficient data in the titles and the respective abstracts to make a clear decision, the full texts of the articles were retrieved for further analysis. The final inclusion of the relevant full-text articles for evaluation was decided by consensus between the same two observers. (K.S.Y and D.R)

### 2.4 Data extraction process

A “selected studies table” (S2 Table) was constructed that included only the studies from which measurements could be extracted. This table included the following: author(s) name, title, year of publication, study aim, study population data (no. of patients, sex, age), study measurement tools (CBCT, in-vitro, in-vivo etc.), site characteristics (dentate/edentulous, jaw), site location (anterior/premolar/posterior or tooth position (incisor/premolar/molar)), site measurement (ridge width/ buccal width/palatal or lingual width), distance from alveolar ridge crest, statistical data provided as well as main findings.

**Fig 1:**
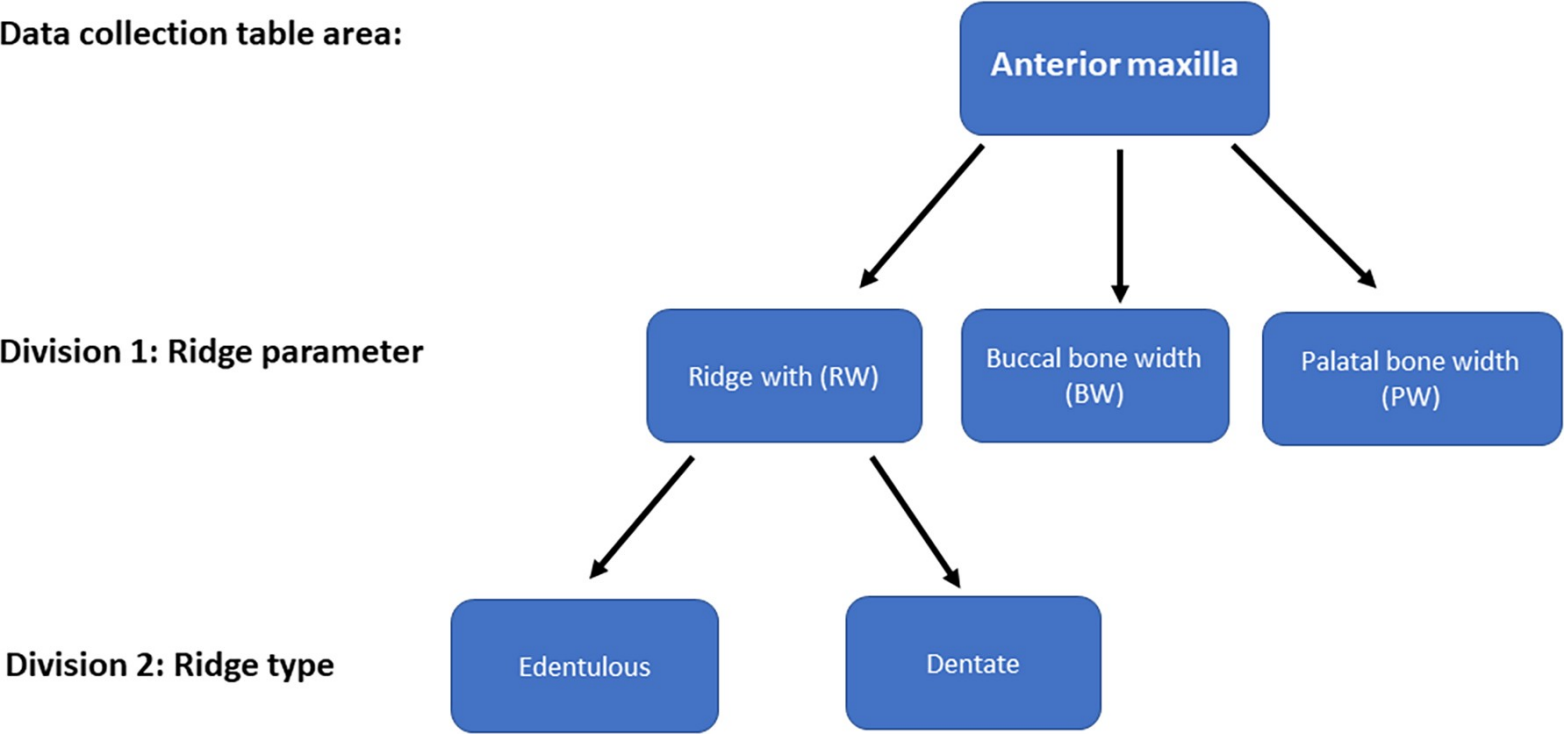
Elaboration process scheme.

From the selected studies, linear measurements data tables were constructed. Each of the 6 tables corresponds to a different area of the jaw (anterior maxilla, premolar maxilla, posterior maxilla, anterior mandible, premolar mandible, posterior mandible). Each table was subdivided into separate tables which correspond to the measured parameter (ridge width / buccal width / palatal or lingual width), reaching a total of 18 tables. Each table was divided again into final data tables according to site characteristics (dentate and edentulous). Each table contains the name of the study included, and the measurement obtained from the study (mean+standard deviation, number of measurements included) and the distance from the alveolar crest where the measurement was done. The table elaboration process is shown schematically in Fig 1.

### 2.5 Quality assessment

The assessment of study quality was performed for all the included articles based on the JBI checklist [28]. Because no randomized control trials (RCT) were included, we based our checklist on the recommendations applying to ‘Quasi Experimental Study”, with adequate modifications to fit exactly into the context of the present research (see S1 File). The available answers were “yes”, “no”, “unclear”. The risk of bias was assessed independently by the two above-mentioned examiners.

### 2.6 Data analysis

The quantitative construction of the alveolar ridge from the obtained data was done by analyzing the combined mean of each component of the alveolar ridge for every millimeter from the crest. In order to find the combined mean with the 95% confidence interval from all studies included in the analysis, we used meta-analysis (when at least two studies with relevant data per outcome were available). In all the analyses done, we used the random effects model. We assessed statistical heterogeneity using I^2^ and Q statistics Meta-analyses were performed separately for every combination of size, width, position and dentate/edentulous.

In addition, further meta regression statistical tests were performed to identify differences in outcome parameters for each millimeter from the crest, and differences between the different components of the alveolar ridge. Post-hoc tests were performed when comparison was done between more than two outcome parameters.

## 3. Results

### 3.1 Study selection

**Fig 2:**
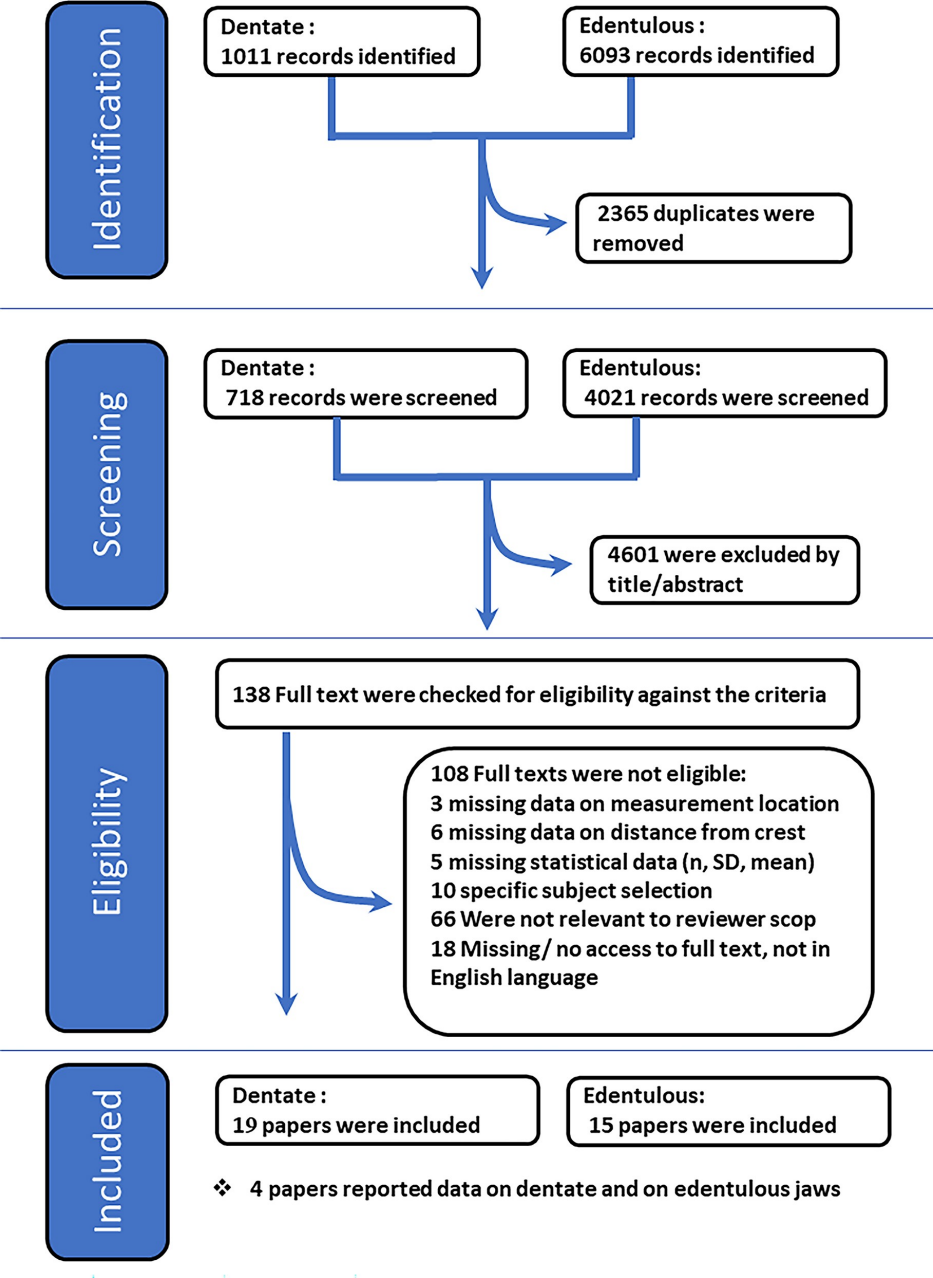
Search and article selection process.

A total of 7104 studies were identified through the electronic search.1011 records dealt with dentate alveolar ridges and 6093 on edentulous. After removing duplicates, a thorough screening of bibliographies of the relevant included/excluded articles yielded a total of 4739 citations (Fig 2).

Upon exclusion of 4601 publications based on their titles/abstract, 138 studies remained for full-text evaluation. Finally, based on full text assessment, an additional 108 studies were excluded yielding 30 studies with quantitative data, of which 19 provided data on dentate alveolar ridge and 15 on edentulous alveolar ridge, noting that 4 reported data on both dentate and edentulous, (Fig 2).

### 3.2 Study characteristics

The characteristics of the 30 included studies are listed in S1 Table. The majority of the selected studies (total of 27) used living human live subjects to analyze alveolar ridge dimensions and its parameters [17, 29–54], 2 studies used cadaver heads [24, 55] and 1 study used dry skulls [56]. The most frequent analysis tool was the CBCT, with measurements extracted using a proprietary software to identify the alveolar ridge parameters and measure their dimensions. The remaining in-vivo/ in-vitro studies measured these parameters using a caliper or a microscope ruler.

Results about the mandible were collected from 18 studies, of which 11 were dentate jaws and 7 were edentulous. Six studies examined the anterior mandible, 14 examined premolar area and 14 posterior area of the mandible. Results about the maxilla were collected from 26 studies, of which 17 were for dentate jaws and 9 for edentulous. Fourteen studies examined the anterior maxilla, 14 examined premolar area and 13 posterior parts of the maxilla.

The measured dimensions outcome, i.e. RW, BW, PW/LW, and CaW (cancellous bone width), were extracted from 21, 16, 7 and 1 studies, respectively.

### 3.3 Quality assessment

**Fig 3:**
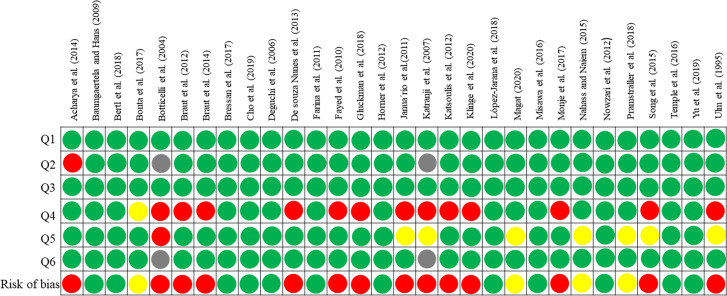
Risk of bias assessment for the included studies. Green indicates low risk, yellow-medium and red-high risk. Grey means undefined.

The result of the risk of bias assessment for the included studies is presented in Fig 3. 12 articles were considered as low risk, 4 studies were considered as moderate risk and 14 studies were considered as high risk.

### 3.4 Synthesis of results and meta-analysis

The combined mean was calculated along the occlusal-apical direction of the ridge in one-millimeter intervals (dentate and edentulous) both for the total ridge width (RW) and the two cortical walls (BW and LW/PW). The results are presented in Table 1 for the maxilla and in Table 2 for the mandible. The tables present, for each millimeter along the alveolar ridge the number of studies included in the analysis, the calculated combined mean with the 95% confidence interval and the consistency (I^2^).

**Table 1:** Meta-analysis result for the maxilla.

			Anterior maxilla			Premolar maxilla			Posterior maxilla		
State	parameter	mm	No. study Groups	mean (95%CI)	I^2^	No. study Groups	mean (95%CI)	I^2^	No. study Groups	mean (95%CI)	I^2^
**Dentate**	**BW**	**1**	17	0.73 (0.65 to 0.77)	96.9%	6	0.96 (0.69 to 1.23)	93.8%	5	1.25 (1.02 to 1.48)	97.6%
		**2**	9	0.97 (0.88 to 1.06)	96.2%	8	1.16 (1.13 to 1.2)	49.4%	10	1.18 (1.1 to 1.26)	87.4%
		**3**	7	0.83 (0.66 to 1)	98.5%	5	1.08 (0.71 to 1.44)	95.7%	5	1.49 (1.06 to 1.91)	98.8%
		**4**	12	1.01 (0.94 to 1.09)	95.4%	9	1.12(1.05 to 1.2)	86.0%	14	1.2 (1.11 to 1.29)	91.9%
		**5**	6	0.72 (0.54 to 0.89)	98.8%	8	1.08 (0.85 to 1.31)	97.3%	8	1.23 (0.98 to 1.48)	97.8%
		**6**	7	1.11 (1.06 60 1.16)	79.7%	8	1.23 (1.18 to 1.29)	66.8%	10	1.27 (1.21 to 1.33)	78.7%
	**LW/PW**	**1**	6	0.88 (0.78 to 0.97)	72.0%	3	0.99 (0.28 to 1.69)	93.1%	ND	ND	ND
		**2**	5	1.46 (1.3 to 1.61)	87.8%	4	1.57 (1.5 to 1.65)	50.0%	4	1.39 (1.38 to 1.40)	0.0%
		**3**	3	1.69 (1.3 to 2.08)	68.3%	3	1.75 (1.33 to 2.17)	83.6%	ND	ND	ND
		**4**	5	1.64 (1.56 to 1.73)	57.6%	4	1.68 (1.57 to 1.78)	68.0%	7	1.47 (1.37 to 1.56)	74.0%
		**5**	2	2.24 (1.75 to 2.73)	48.8%	6	1.88 (1.64 to 2.12)	91.7%	4	1.56 (1.31 to 1.8)	92.1%
		**6**	5	1.75 (1.73 to1.78)	0.0%	4	1.72 (1.66 to 1.77)	0.0%	4	1.49 (1.4 to 1.57)	75.6%
	**RW**	**1**	10	6.39 (5.45 to 7.32)	99.0%	9	7.97 (6.54 to 9.40)	99.1%	6	9.96 (7.83 to 12.08)	99.4%
		**2**	5	7.85 (7.46 to 8.24)	81.7%	4	9.32 (8.88 to 9.77)	85.7%	4	12.11 (11.24 to 12.98)	96.2%
		**3**	7	7.23 (6.78 to 7.68)	94.0%	7	8.76 (8.1 to 9.41)	93.3%	3	12.31(11.62 to 13.01)	56.7%
		**4**	5	8.55 (8.2 to 8.9)	73.0%	4	9.74 (9.34 to 10.14)	78.5%	4	12.78 (11.88 to 13.67)	96.0%
		**5**	2	8.64 (7.76 to 9.52)	75.3%	6	9.6 (9.01 to 10.19)	87.8%	4	13.2 (11.96 to 14.44)	95.2%
		**6**	9	8.34 (7.71 to 8.96)	96.0%	6	9.53 (8.85 to 10.22)	94.9%	4	13.42 (12.51 to 14.33)	95.0%
**Edentulous**	**RW**	**1**	3	3.36 (2.91 to 3.82)	0.0%	5	4.47 (3.47 to 5.48)	78.4%	17	5.6 (4.88 to 6.32)	94.9%
		**2**	ND	ND	ND	6	7.24 (6.49 to 7.98)	87.5%	6	8.51 (7.91 to 9.11)	75.4%
		**3**	6	3.87 (3.61 to 4.14)	17.3%	7	5.56 (4.53 to 6.59)	94.0%	18	7.79 (7.14 to 8.45)	91.0%
		**4**	ND	ND	ND	ND	ND	ND	ND	ND	ND
		**5**	2	5.12 (5.04 to 5.2)	0.0%	2	6.77 (5.4 to 8.14)	67.1%	ND	ND	ND
		**6**	ND	ND	ND	ND	ND	ND	ND	ND	ND

The table includes, for each millimeter along the alveolar ridge, according to the ridge parameter in the studied area (RW, BW and PW), the number of studies included in the analysis, the calculated combined mean with the 95% confidence interval and the consistency (I^2^). ND indicates insufficient data to perform a meta-analysis.

**Table 2:** Meta-analysis result for the mandible.

			Anterior mandible			Premolar mandible			Posterior mandible		
State	parameter	mm	No. study Groups	mean (95%CI)	I^2^	No. study Groups	mean (95%CI)	I^2^	No. study Groups	mean (95%CI)	I^2^
**Dentate**	**BW**	**1**	ND	ND	ND	4	0.78 (0.55 to 1.01)	93.4%	5	0.78 (0.62 to 0.95)	75.8%
		**2**	6	1.09 (0.93 to 1.25)	96.5%	10	0.97 (0.68 to 1.27)	99.1%	14	1.57 (1.24 to 1.89)	96.8%
		**3**	ND	ND	ND	3	0.77 (0.41 to 1.14)	93.5%	5	1.61 (1.06 to 2.15)	97.3%
		**4**	7	1.11 (1.02 to 1.2)	87.5%	9	1.4 (1.28 to 1.52)	88.8%	11	2.31 (2.01 to 2.61)	94.5%
		**5**	ND	ND	ND	6	1.19 (0.93 to 1.45)	96.0%	8	2.47(1.76 to 3.19)	99.3%
		**6**	6	1.17 (1.13 to 1.22)	52.8%	8	1.61 (1.47 to 1.75)	88.7%	10	2.59 (2.29 to 2.88)	94.3%
	**LW/PW**	**1**	ND	ND	ND	ND	ND	ND	ND	ND	ND
		**2**	5	1.79 (1.63 to 1.95)	81.2%	6	1.85 (1.42 to 2.28)	95.0%	8	1.9 (1.84 to 1.96)	0.0%
		**3**	ND	ND	ND	ND	ND	ND	ND	ND	ND
		**4**	5	2.1 (1.94 to 2.26)	77.9%	4	2.53 (2.42 to 2.65)	28.6%	7	2.09 (2 to 2.19)	25.3%
		**5**	ND	ND	ND	4	2.77 (2.49 to 3.05)	76.4%	4	2.5 (2.27 to 2.73)	85.2%
		**6**	5	2.22 (2.13 to 2.31)	24.4%	4	2.49 (2.44 to 2.54)	0.0%	4	2.32 (2.23 to 2.42)	10.6%
	**RW**	**1**	7	5.67 (4.66 to 6.68)	99.0%	8	6.95 (5.76 to 8.14)	98.8%	10	9.33 (7.87 to 10.79)	99.4%
		**2**	5	6.99 (6.58 to 7.4)	78.8%	4	8.39 (8.17 to 8.61)	0.0%	4	11.2 (9.88 to 12.51)	96.7%
		**3**	ND	ND	ND	2	8.75 (7.87 to 9.63)	81.6%	3	10.73 (9.34 to 12.12)	97.2%
		**4**	5	7.27 (6.85 to 7.68)	81.8%	4	9.09 (8.85 to 9.33)	4.7%	4	11.94 (10.67 to 13.21)	95.9%
		**5**	ND	ND	ND	5	9.64 (8.69 to 10.58)	88.5%	6	12.68 (11.67 to 13.58)	96.0%
		**6**	5	7.42 (7.13 to 7.72)	51.5%	4	9.47 (9.08 to 9.86)	60.5%	4	12.48 (11.37 to 13.58)	94.5%
**Edentulous**	**RW**	**1**	ND	ND	ND	5	4.08 (3.5 to 4.66)	85.2%	8	5.6 (4.5 to 6.71)	97.6%
		**2**	ND	ND	ND	ND	ND	ND	ND	ND	ND
		**3**	ND	ND	ND	4	6.89 (6.43 to 7.34)	62.3%	7	8.45 (7.47 to 9.43)	94.5%
		**4**	ND	ND	ND	2	5.88 (5.43 to 6.33)	8.2%	2	8.11 (7.18 to 9.04)	69.3%
		**5**	ND	ND	ND	2	9.11 (8.76 to 9.46)	7.7%	5	11.37 (10.65 to 12.09)	90.8%
		**6**	ND	ND	ND	ND	ND	ND	ND	ND	ND

The table includes, for each millimeter along the alveolar ridge, according to the ridge parameter in the studied area (RW, BW and LW), the number of studies included in the analysis, the calculated combined mean with the 95% confidence interval and the consistency (I^2^). ND indicates insufficient data to perform a meta-analysis.

**Fig 4:**
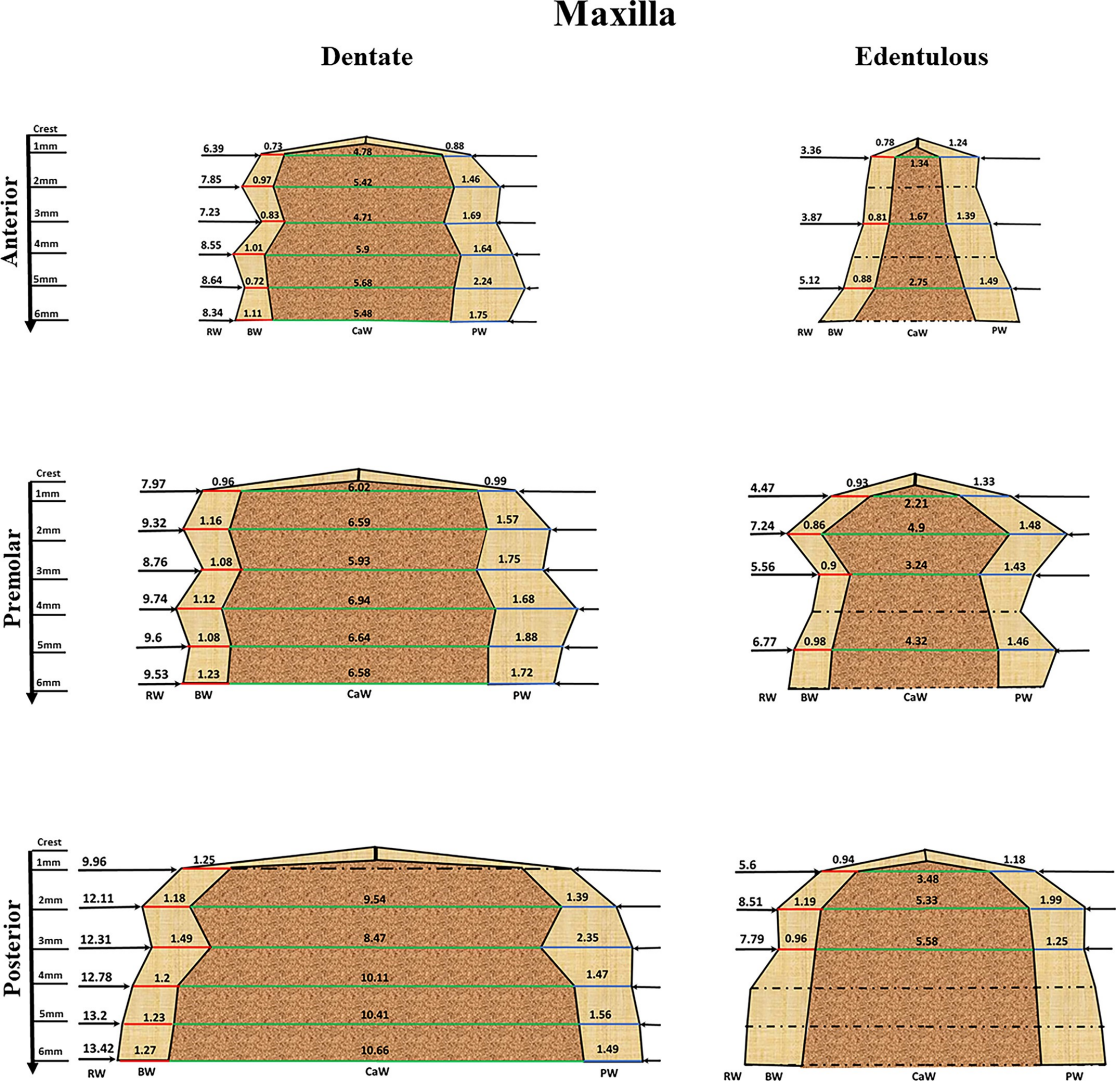
The maxillary dentate and edentulous ridge. The fig was constructed by using the combined mean obtained from the meta-analysis results for the RW (Ridge width) marked by a black line. BW (Buccal width) marked by a red line and PW (Palatal width) marked by a blue line and the calculated CaW marked by green line. The interrupted lines represent an estimated width.

**Fig 5:**
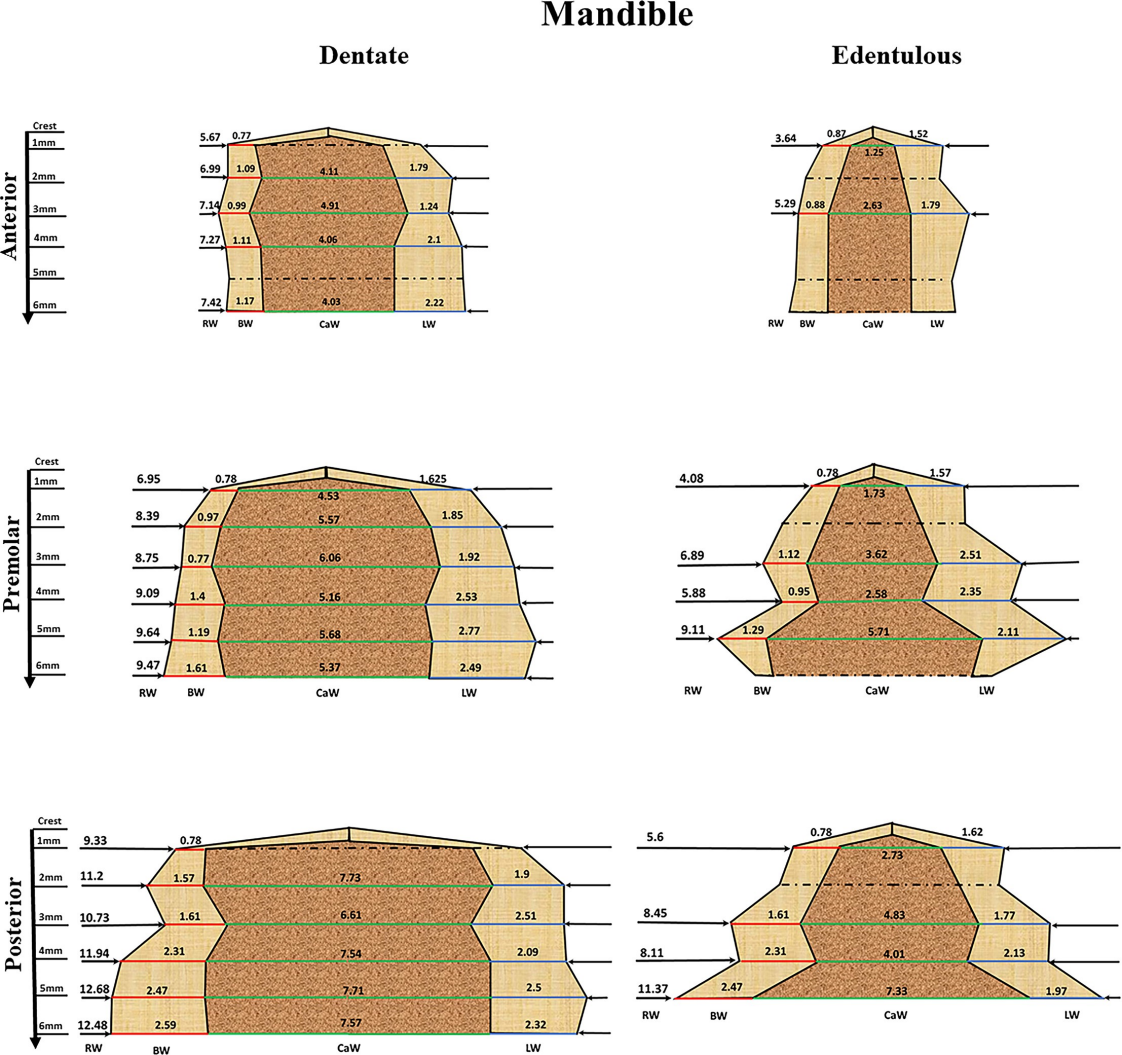
The mandible dentate and edentulous ridge. The fig was constructed by using the combined mean obtained from the meta-analysis results for the RW- black line, BW—red line and LW—blue line and the calculated CaW marked by green line. The interrupted lines represent an estimated width.

We used the calculated combined mean to construct a representative alveolar ridge in the maxilla (Fig 4), and in the mandible (Fig 5). Due to lack of relevant data, only the first 6 mm (vertical dimension) along the alveolar ridge were used for the analysis, in both dentate and edentulous ridges. In addition, data on BW and PW/LW in edentulous ridges are missing in the literature, so that in order to reconstruct the edentulous ridges, we used 75% of the BW combined means of the dentate parameters and 80% of PW/LW according to the earlier mentioned reported bone resorption subsequent to tooth extraction [23].

### 3.5 Assessment of alveolar ridge components and their inter-relations

A comparison between the different millimeters of the same outcome parameter (1mm vs. 2mm vs.3mm vs. 4mm etc.) in the maxilla and mandible revealed that along the 6 mm of the alveolar crest no meaningful differences were observed. The only statistical difference was found between the first 1mm of the alveolar ridge and the following millimeter in BW (95%CI = 0.11–0.4, P<0.01) and PW (95%CI = 0.42–0.73, P<0.01) of the anterior maxilla.

The alveolar ridge width (RW, Tables 1 and 2) in the dentate maxilla shows a steady significant increase, from anterior areas to premolar areas (95%CI = 0.75–2.04, P<0.0001) and posterior areas. (95%CI = 2.35–3.8, P<0.0001). The same behavior was observed in the mandible were RW increased significantly from anterior areas, premolar areas (95%CI = 0.81–2.57, P<0.0001) to posterior areas (95%CI = 1.83–3.45, P<0.0001).

By contrast, the calculated combined mean RW of the edentulous alveolar ridges shows a clear and pronounced reduction, between 17%-57% in the maxilla and between 5–50% in the mandible, with a more noticeable reduction in crestal parts of the ridge that can exceed 50%. No difference was observed in RW reduction between the different areas of the Jaws.

Buccal bone width (BW) in the anterior maxilla was found to be statistically different from BW in premolar area (95%CI = 0.14–0.33, P<0.01), and posterior maxilla (95%CI = 0.27–0.45, P<0.001). No statistical difference was found between the premolar maxilla and posterior maxilla BW (95%CI = 0.02–0.21, P = 0.015). In the mandible, the anterior and the premolar areas shows a comparable outcome (95%CI = (-0.25)-0.43, P = 0.625). But BW at the posterior area was found to be significantly larger than in the anterior (95%CI = 0.53–1.2ת P<0.001) and premolar areas (95%CI = 0.51–1.03 P<0.001).

Palatal cortical bone (PW) in the anterior maxilla was found to be statistically different from PW in the premolar area (95%CI = 0.75–2.05, P<0.001) and the posterior area (95%CI = 3.76–5.19, P<0.001). In addition, PW in the premolar area was found to be statistically different to PW in posterior area (95%CI = 2.35–3.8, P<0.001).

Lingual cortical bone (LW) in the anterior mandible, was found statistically different from LW in premolar area (95%CI = 0.08–0.54, P = 0.0074). No statistical difference was found between LW in anterior area and posterior area (95%CI = (-0.05)-0.38, 0.08–0.54, P = 0.14) and premolar area and posterior area (95%CI = (-0.06)-0.36, P = 0.17)

From a comparison between BW and PW in the maxilla, it seems that BW is narrower than PW. The difference was found to be statistically meaningful in anterior areas (95%CI = 0.47–0.74, P<0.001), premolar area (95%CI = 0.40–0.66,P<0.001) and posterior area (95%CI = 0.12–0.36,P<0.001). In the mandible, BW was narrower than LW in all the evaluated areas. The difference was found to be statistically meaningful in anterior areas (95%CI = 0.8–1.03, P<0.001), premolar area (95%CI = 0.89–1.39, P<0.001), but not in posterior area (95%CI = (-0.14)-0.57, P<0.001).

### 3.6 Bone classification in dentate and edentulous ridges

Based on the quantitative data (section 3.4 and Tables 1 and 2), different bone types can now be defined in the spirit of Lekholm and Zarb [3]. Using the combined mean width of alveolar ridge (RW) and the relative parts of the cortical components, the bone type can now be defined as:

$$Bone\;type\;(\%\;of\;cortical\;components\;of\;total\;ridge\;witdh)\;=\;\frac{\left(BW+PWorLW\right)}{RW}\times 100$$

**Bone type 1**: The relative part of the cortical bone (Buccal bone + Palatal/Lingual bone) in the total ridge width exceeds 75%.

**Bone type 2**: The relative part of the cortical bone in the total ridge width lies in the range 50–75%.

**Bone type 3**: The relative part of the cortical bone in the total ridge width lies in the range 25–50%.

**Bone type 4**: The relative part of the cortical bone in the total ridge width is inferior to 25%.

Table 3 lists the calculated bone types for the different areas of the maxilla and mandible from the above-described quantitative analysis. The table further describes Misch’s classification for bone types in edentulous ridges according to the different areas of the maxilla and mandible, as well as Chatvaratthana et al’s. [6] quantitative bone classification for comparison.

**Table 3:** Bone types analysis.

Site	state	mean Cor/RW%	Bone type	Bone type (according to Misch 1988)	mean CorW/CanW%	Bone type (according to Chatvaratthana et al. 2017)
Anterior maxilla	Dentate	31.8%	3		46.9%	3
	Edentulous	54.4%	2	2 or 3	95.4%	1
Premolar maxilla	Dentate	29.4%	3		41.9%	3
	Edentulous	47.0%	3	3	69.7%	2
Posterior maxilla	Dentate	23.0%	4		30.3%	3
	Edentulous	34.6%	3	3 or 4	53.5%	2
Anterior mandible	Dentate	40.6%	3		77.75%	1
	Edentulous	58.0%	2	1 or 2	>100%	1
Premolar mandible	Dentate	37.8%	3		71.9%	2
	Edentulous	50.0%	2	2	87.5%	1
Posterior mandible	Dentate	37.0%	3		58.1%	2
	Edentulous	45.1%	3	3	82.5%	1

The table describe the calculated mean Cor/RW% for the different areas of the maxilla and mandible in dentate/edentulous state and the corresponding bone type. The table further describes for comparison Misch’s classification for bone types, only in edentulous ridges and the calculated mean Cor/Can% according to Chatvaratthana et al’s. [6] quantitative bone classification for comparison.

This analysis shows that the dentate alveolar bone (maxilla and mandible) can be classified as bone type 3 with a relative part of cortical bone between 29.4% to 40.6% in most areas (except for the posterior maxilla where the relative part is 23%). The presence of teeth with the supporting apparatus (PDL, alveolar bone proper) preserves the bone volume and the relative dimensions of the cortical and trabecular bone.

In edentulous ridges, the bone type changes to 2 in the anterior maxilla, anterior mandible and premolar mandible with a relative part of cortical bone of 54.4%, 58% and 50%, respectively. In the premolar maxilla, posterior maxilla and posterior mandible, that are the more posterior areas of the jaws, the bone type remains 3 with a relative part of cortical bone of 40.2%, 34.6% and 45.1%, respectively.

## 4. Discussion

The present work aims at refining our understanding of the bone type classification that has been essentially qualitative so far. For this purpose, information was first gathered on the alveolar ridge components in different areas of the jaws, in both dentate and edentulous states.

It was found that along the 6 mm of the alveolar crest, there are no meaningful differences in RW, BW and PW/LW. This finding contradicts most published data reporting a consistent increase in the RW [31] BW and PW/LW in the dentate maxilla and the mandible, when moving apically [31, 37, 39, 56, 57]. This happens probably since statistical comparisons were performed between consecutive depths along the ridge and not between intervals/ group of depths.

Regarding the different areas of the jaws, the outcomes of our study show a continuous increase in RW from anterior to posterior areas of both the dentate maxilla and mandible. This finding is in accordance with other studies, both in dentate [46, 58] and in edentulous jaws [35, 40, 50, 54]. A similar trend was shown for BW and PW/LW, as in [31, 49], which clearly outlines and support the importance of *defining different areas of the jaws*.

Another consistent finding in all included studies, comparing dentate and edentulous RW’s, is the clear reduction in RW after tooth extraction. This reduction was found to be more meaningful in crestal areas of the ridge, where RW reduction exceeds 50% [30, 39, 59]. This finding is in agreement with studies evaluating the effect of buccal cortical bone width on the post extraction horizontal changes [18–21]. In areas of the jaw where buccal bone width was less than 1mm (e.g., the anterior maxilla), the amount of reduction in RW exceeded 50%. On the opposing side, where BW was larger than 1mm (e.g., the posterior maxilla, posterior mandible apical parts of the ridge), the amount of reduction in RW was inferior to 35%.

We propose here a slightly different quantitative classification, in which the denominator is modified to include the total ridge dimension, RW. As shown in Table 3, this modification yields a good correlation and almost full agreement with the qualitative classification of bone type according to specific area of the jaw proposed by Misch. Note that if we interpret the results according to Chatvaratthana et al. [6], although that approach is questioned by [60, 61], all the edentulous cases are of the 1 or 2 bone type. In other words, the edentulous bone becomes essentially cortical, a point that is debatable from a clinical standpoint.

Our study refines the concept by providing a classification that examines each region of the jawbone separately, a point not directly found in Misch [5] or in Leckholm and Zarb’s [3] classification, all viewing the jaws as one and uniform unit.

One of the outcomes of our work lies in the fact that a relatively accessible geometry-based analysis can yield valuable insights on the bone type, of a kind that might be delicate to determine otherwise, e.g., through local density variations over time. In that respect, it is believed that the results presented are original in that they quantitatively complement the well-established qualitative approach while making it more accessible from a routine procedure.

The substantial heterogeneity found during the meta-analysis is a well-known fact in Cochrane and non-Cochrane meta-analyses, either in dentistry or in medicine [62, 63]. Several factors contribute to this high heterogeneity, which are mostly connected to the study population selected in the included studies. Factors, such as possible anatomical difference between males and females [31, 42, 57–59], possible age effects (young vs. aged population), as in [31, 38, 57], or ethnic differences [29], were all found to effect alveolar ridge components, contributing to statistical heterogeneity. Moreover, no information was given in any of the included studies on the healing time following tooth extraction, which is a determinant factor influencing the extent of ridge resorption and the difference between RW in dentate and edentulous state [15, 16].

Finally, it is noted that this study relies on published results rather than direct clinical measurements, where 90% of the included studies used the CBCT to evaluate alveolar ridge parameters. Although the use of live measurement is more accurate, it lacks the advantage of a large study group and involves significant clinical complexity. The key disadvantage is possible accuracy error related to technical issues [25]. With that, the accuracy of one group measurements will be entirely dependent on the accuracy of the measurements, whereas by gathering data from various sources, this risk is somewhat mitigated or averaged.

## 5. Conclusions

The study identified alveolar bone and its components behavior in dentate and edentulous state.

A continuous increase in RW, BW and PW/LW was observed from anterior to posterior areas of both the dentate maxilla and mandible, which clearly outlines and supports the importance of defining different areas of the jaws.

The study provided a geometry-based bone type classification for each region of the jawbone which was found to be in full agreement with the common qualitative classification.

## Supporting information

S1 Table Search strategy keywords specific for the study question. (DOCX)

S2 Table General characteristics of the included articles. (DOCX)

S1 File PRISMA-P 2015 checklist. (DOCX)
