# Supplementary material for: Quantitative assessment of the jawbone quality classification: A meta-analysis study
Source: PLoS One. 2021 Jun 16;16(6):e0253283. doi: 10.1371/journal.pone.0253283 (PMC8208540; doi:10.1371/journal.pone.0253283)
Supplement: S1 Table — (DOCX) [file pone.0253283.s001.docx]

|  | **term 1** | | **term 2** | | **term 3** |
| --- | --- | --- | --- | --- | --- |
|  | **Edentulous** | **Dentate** | **Alveolar Process** | **Maxilla/Mandible** | **Dimension** |
| **MeSH** | Mouth, Edentulous |  | Alveolar Process | Mandible |  |
|  | Jaw, Edentulous |  | Tooth Socket | Maxilla |  |
|  | Jaw, Edentulous, Partially |  |  |  |  |
| **Emtree** | Edentulousness |  | Alveolar ridge | Mandible |  |
|  |  |  |  | Maxilla |  |
| **Text words** | Toothless Mouth | Dentate | Alveolar Processes | Maxillofacial | Dimensional |
|  | Edentulous |  | Alveolar Process | Maxillae | Dimension |
|  |  |  | Alveolar Ridge | Maxillas | Dimensions |
|  |  |  | Ridge | Maxillary | Width |
|  |  |  |  | Maxillar | Thickness |
|  |  |  | Cortical Bone | Maxillaris | Alterations |
|  |  |  | Cortical wall | Mandibular | Alteration |
|  |  |  | Trabecular bone | Mandibles | Morphology |
|  |  |  | Cancellous bone | Mandible | Morphologic |
|  |  |  | Alveolar bone |  | Anatomy |
|  |  |  | Facial bone |  | Anatomical |
|  |  |  | Bone wall | Crestal | Microarchitecture |
|  |  |  |  | Bucco-palatal | Height |
|  |  |  |  | Buccolingual | Radiomorphometric |
|  |  |  |  | Buccal | Depths |
|  |  |  |  | Palatal | Morphometric |
|  |  |  |  | Lingual | Anthropometric |

**S1 Table: Search strategy keywords specific for the study question**
