# Supplementary material for: Quantitative assessment of the jawbone quality classification: A meta-analysis study
Source: PLoS One. 2021 Jun 16;16(6):e0253283. doi: 10.1371/journal.pone.0253283 (PMC8208540; doi:10.1371/journal.pone.0253283)
Supplement: S2 Table — (DOCX) [file pone.0253283.s002.docx]

| Article | Study aim | Study group | | | Measurements  methods | Location | | Parameter | | Stat. data | Main conclusion |
| --- | --- | --- | --- | --- | --- | --- | --- | --- | --- | --- | --- |
|  |  | No. of subjects | Sex | Age  Mean (Range) |  | Jaw | Location (total no. of sites) |  | Mm from crest |  |  |
| Acharya et al. (2014) | Residual ridge dimensions at  edentulous maxillary first molar sites  and periodontal bone loss among two  ethnic cohorts | 628 live | F+M | 52 (NR) | CBCT measurements were done using a  software program | Ed. Max. | M1 (628) | RW | 1,3 | Mean + SD  Median+IR | Gender and the presence or the absence of  adjacent teeth affected the buccolingual ridge  width. |
| Baumgaertela and Hans (2009) | To investigate the buccal cortical bone thickness of every interdental area | 30 dry skulls | NR | NR | CBCT measurements were done using a  software program | D. Max.  &  Mand. | C, LI, CI (300) | BW | 2, 4, 6 | Mean+  SD | BW is larger in the mand. then max. BW is thinnest in the anterior sextants of both jaws and  increases progressively toward the posterior, except for the tuberosity area |
| Bertl et al. (2018) | To assess Sinus width at different height levels of posterior teeth. residual alveolar ridge dimensions were evaluated as well | 162 live | F+M | 55(NR) | CBCT measurements were done using a  software program | Ed. Max. | PM (138)  M (224) | RW | 2 | Mean +SD | RW was significantly larger in the molar region compared to the premolar region |
| Bonta et al. (2017) | To evaluate BW morphology related to the tooth position in the alveolar crest in the maxillary anterior area | 54 live | F+M | 41.5 (18-65) | CBCT measurements were done using a  software program | D. Max. | CI LI and C (203) | BW | 1 | Mean SD Median Max, Min | Weak negative correlations were found between facial position tooth and the thickness of the facial bone wall at 1mm |
| Botticelli et al. (2004) | To study dimensional alterations of hard tissues that occur following tooth extraction. | 18 live | F+M | 49.1 (21-81) | In-vivo, measured during treatment with a caliper | D. Max. & Mand | CI, LI, C (5)  PM1 (16) | RW, BW L/PW | 1 | Mean+  SD | The width of the bone wall of the extraction socket was lingually then buccally. The horizontal resorption of the bone crest at the buccal site was larger then in the lingual site. |
| Braut et al. (2012). | To analyze the dimensions of the alveolar  bone in the posterior dentate mandible | 56 live | F+M | 54.5 (17-82) | CBCT measurements were done using a  software program | D. Mand. | PM1, PM2 (82)  M1, M2 (80) | BW, L/PW and RW | 1, 2 | Min, Max, Median, Mean, Q1 and Q3 | There was a steady increase in BW from PM1 to M2. RW at the level of the PM was significantly thinner than that for M. |
| Braut et al. (2014) | To analyze dimensions and morphology of edentulous sites in posterior mand. | 56 live | F+M | 54.5 (17-82) | CBCT measurements were done using a  software program | Ed. Mand. | PM1, PM2 (54)  M1, M2 (72) | RW | 1,4 | Min, Max, Mean, Median | There was an increase in RW from PM1 to M2. The majority of sites demonstrated an increase of width in an apical direction and measure more them 6 mm at 4mm from crest |
| Bressan et al. (2017) | To evaluate ridge dimensions of posterior sextant in totally edentulous mandibles. | 136 live | F+M | 67.4 (27-92) | CBCT measurements weredone using a  software program | Ed. Mand. | PM2 (385)  M1, M2 (769) | RW | 1, 3, 5 | Mean, Median, SD, Min, Max | RW and BW increased from PM to M, at all height levels with no difference in BW between M and F and different age. |
| Cho et al. (2019) | To evaluate the normal alveolar bone structure for use in diagnosing for implant placement | 22 live | F+M | 23 (20-29) | CBCT measurements were done using a  software program | D. Max. & Mand. | CI, C (132)  PM2 (66)  M1 (66) | RW | 1 | Mean+  SD | The BW is very thin in the anterior upper jaw. RW in the molar area was larger in the Max> Mand |
| Deguchi et al. (2006) | To evaluate cortical bone thickness in various locations in the maxilla and the mandible | 10 live | F+M | 22.3 (NR) | CT measurements were done using a  software program | D. Max. & Mand. | M2, M1 (100) | BW L/PW | 4, 7 | Mean+  SD | BW increased from M2 to M1. No increase in width in an apical direction. |
| De souza Nunes et al. (2013) | To analyze the width and height of edentulous sites in posterior maxilla. | 122 live | F+M | 57.5 (21-92) | CBCT measurements were done using a  software program | Ed. Max. | PM1, PM2 (111)  M1, M2 (141) | RW | 2 | Mean Max Min 95%CL | The trend showed that the more distally the site was located the larger RW. Sex and Age did not affect the results. |
| Farina et al. (2011) | To analyze alveolar ridge dimensions in maxillary  posterior sextants of dentate and  edentulous sites | 32 live | F+M | 55.9 (32-71) | CBCT measurements were done using a  software program | D. and Ed. Max. | PM (42)  M (64) | RW | 1, 3, 7 | Mean+SD | Edentulous sites showed significantly lower RW compared with dentate sites |
| Fayed et al. (2010) | Dimensional mapping of the interradicular spaces and cortical bone thickness.  The effect of age and sex was evaluated | 100 live | F+M | 20 (13-27) | CBCT measurements were done using a  software program | D. Max. & Mand. | M2, M1 (528)  PM1, PM2 (528)  C, LI and CI (660) | RW, BW L/PW | 2, 4, 6 | Mean+  SD | The results of this study showed a consistent increase in the RW BW and LW/PW thickness in most of the studied sites in the maxilla and the mandible when moving apically and posteriorly. |
| Gluckman et al. (2018) | To introduce a new classification system for anterior maxilla tooth position. Data for facial and palatal bone wall height and thickness are also presented | 150 live | F+M | 49.4 (18-89) | CBCT measurements were done using a  software program | D. Max. | CI (233)  LI (175)  C (183) | BW PW | 1 | Mean + SD | BW with was <1mm at the crest and apically. Most palatal walls were <1mm at the crest and >1mm apically. |
| Horner et al. (2012) | To assess differences in dento-alveolar cortical bone thickness between  hyperdivergent and hypodivergent young adults | 57 live | F+M | 27.5 (NR) | CBCT measurements were done using a  software program | D. Max. & Mand. | M2, M1 (228)  PM1, PM2 (228) | BW  L/PW  RW CanW | 5 | Mean+  SD | The PW of the maxilla was consistently thicker than the BW, with all sites. In each region, the posterior BW tended to have thicker cortical bone than did the anterior sites. The anterior lingual sites were significantly thicker than the posterior lingual site. Mandibular cortical bone was significantly thicker than maxillary cortical bone |
| Janua´rio et al.(2011) | To determine the thickness of the BW in the anterior dentition of the maxilla  and at different locations apical to the (CEJ). | 250 live | F+M | 37.9 (17-66) | CBCT measurements were done using a  software program | D. Max. | CI (500)  LI (500)  C (500) | BW | 1, 3, 5 | Mean+  SD | Most tooth sites in the anterior maxilla have a thin BW (<1mm) the thickness of the BW did not vary with age or the location of measurements |
| Katranji et al. (2007). | To determine an average cortical bone thickness in different  tooth locations. | 28 cadaver heads | F+M | NR (73.1) | In-vivo, measurements were done with a digital caliper | D. and Ed. Max. &  Mand. | M (44)  PM (44)  C, LI, CI (44) | BW L/PW and RW | 1, 3 | Mean+  SD | The anterior and premolar regions of the dentate maxilla had a greater cortical bone thickness than the mandible. the edentulous mandible has thicker cortical plates compared to the maxilla. |
| Katsoulis et al. (2012) | To analyze bone width in relation to ridge thickness comparing the anterior with the posterior  edentulous maxilla. | 52 live | F+M | 62 (NR) | CBCT measurements were done using a  software program | Ed. Max. | C, LI and CI (156)  PM (208)  M (104) | RW | 3,8 | Mean, Median, SD, 95% CI, Min Max | Mean complete ridge thickness and mean bone width increased significantly from anterior to posterior positions. No differences were observed between female and male subjects |
| Klinge et al. (2020) | To analyze Craniofacial Height in relation to cross-sectional morphology of anterior maxilla: | 180 live | F+M | NR | CBCT measurements were done using a  software program | D. Max. | LI, C (240)  PM1 (120) | RW | 3, 6, 9,12 | Mean + SD  Max-min | At 3 and 6 mm from the crest no significant difference between groups. RW is wider in male in anterior parts of the maxilla on all measured regions |
| López-Jarana et al. (2018) | To anatomically describe the bone morphology in the maxillary and  mandibular tooth areas | 49 live | F+M | 40.3 (NR) | CBCT measurements were done using a  software program | D. Max.  & Mand. | Maxilla:  CI, LI, C (99)  PM (50)  M (59)  Mand:  CI, LI, C (112)  PM (62)  M (21) | BW | 1, 4 | Medium+ SD | In the maxilla and in the mandible, BW of PM and M was the thickest  BW increases from coronal to apical and from the midline to the canine. 53% of incisors presented a thickness of the buccal wall bone less than 1 mm. |
| Magat (2020) | An analysis of edentulous posterior mandibular ridges in the first molar region | 163 live | F+M | 40.37(18-68) | CBCT measurements were done using a  software program | Ed. Mand. | M1 (163) | RW | 1 | Mean SD Min Max | No significant differences were found in RW between the sexes but negative correlations were found between age. |
| Misawa et al. (2016) | To determine dimensional alterations  that occur in Ant. And PM maxilla  following tooth removal. | 69 live | F+M | 40 (23-67) | CBCT measurements were done using a  software program | Ed. & D. Max. | CI, LI, C (46)  PM (23) | BW (D)  P/LW (D)  RW (D+Ed) | 1, 3, 5 | Mean+  SD | In dentate sites BW was thinner in marginal than in more apical levels. Furthermore, the BW was consistently thinner than the PW. the width of the Ed sites was significantly smaller than that of the D sites |
| Monje et al. (2017) | To investigate the influence of posterior mand dimensions at various levels on alveolar bone microarchitecture | 50 live | F+M | 39.55(18-80) | CBCT measurements were done using a  software program | Ed. Mand. | M (50) | RW | 5, 10, 15 | Mean median SD Q1 Q3 | A strong negative  correlation was found on trabecular spacing and ridge width at 5 mm from the crestal-most area |
| El Nahass and Naiem (2015) | Analysis of the dimensions of the  labial bone wall in the anterior  maxilla | 73 live | F+M | 39.6 (24-56) | CBCT measurements were done using a  software program | D. Max. | CI, (73)  LI (73) | BW | 1, 2, 4 | Mean+SD | Males showed higher BW at 4 mm than females at CI and LI . No other difference between males and females at the other distances was identified |
| Nowzari et al. (2012) | To measure BW overlying healthy CI and to determine prevalence of bone thickness <2 mm. | 101 live | F+M | 48.5(15-82) | CBCT measurements were done using a  software program | D. Max. | CI (202) | BW | 1-10 | Min, Max, Median, Mean, SD and mode | The occurrence of >2 mm BW measurements increased with increasing depth. occurrences of >2 mm maxillary BW were found on no more than. 3% of root surfaces 1.0 to 5.0 mm apical to the bone crest. |
| Pramstraller et al. (2018) | To evaluate ridge dimensions at edentulous, mandibular posterior sites, and contralateral dentate sites. | 24 live | F+M | 57.4 (39–72) | CBCT measurements were done using a  software program | D. & Ed. Mand. | PM2 (24)  M (48) | RW | 1, 3, 5 | Mean SD | At all positions, edentulous sites showed a significantly lower RW at 1mm and 3 mm compared to dentate sites. In addition, gender may have a limited impact on the dynamics of ridge resorption following tooth loss. |
| Song et al. (2015) | An analysis of edentulous posterior  mandible for mandibular Body Bone Graft | 40 live | F+M | 52.2 (NR) | CBCT measurements were done using a  software program | Ed. Mand. | PM (20)  M (69) | RW  BW | 3, 5 | Mean SD | The BW at the PM site was statisticaly different apically (>5mm) then to 5 mm of the bone crest but not in M sites |
| Temple et al. (2016) | To evaluate the buccal plate thickness in posterior dentate areas  both the maxilla and mandible | 265 live | F+M | 55.9 (20-85) | CBCT measurements were done using a  software program | D. Max  & Mand. | Maxilla:  PM(310)  M(624)  Mandible:  PM(310)  M(624) | BW | 1, 3, 5 | Geometric mean, 95%CI, min max | Anatomically, the BW in maxilla tends to decrease in thickness apico-coronally whereas, the mand tends to increase in thickness apico-coronally. In Both  jaws an increase in thickness from anterior to posterior. |
| Yu et al. (2019) | To analyze the maxillary sinus anatomy over edentulous ridges in the bilateral posterior maxillary area in Taiwanese patients | 61 live | F+M | 55.3 (25-73) | CBCT measurements done using a  software program | Ed. Max. | PM (32)  M (69) | RW | 2 | Mean SD | A significantly greater RW was observed in the M2 region than in the PM and M1 regions. RW narrower than 6 mm were present only in the PM regions. |
| Ulm et al. (1995) | Study of physical dimension of edentulous maxillary alveolar process in the region of the maxillary sinus. | 47  cadaver heads | F+M | NR (53-94) | Histological sections. measurements were done with Microscope ruler | Ed. Max. | M (94) | RW | 1, 3 | Mean SD | Measurements of the ridge width at 3 mm below the ridge crest showed that the alveolar ridge width became markedly wider in a cranial direction, At 7 and 10 mm below the ridge crest, the width of the alveolar ridge increased to values exceeding 10 mm |

F:female, M: Male, CI: central incisor, LI: Lateral incisor, C: Canine, PM1:first premolar, PM2: Second premolar, M1:first molar, M2: second molar, Ed: edentulous, D: dentate, BW: buccal bone width P/LW: Palatal or lingual bone width, RW: Ridge width, CanW: Cancellous bone width NR: not reported

**S2 Table**: general characteristics of the included articles
